# Psittacosis Outbreak among Workers at Chicken Slaughter Plants, Virginia and Georgia, USA, 2018

**DOI:** 10.3201/eid2511.190703

**Published:** 2019-11

**Authors:** Kelly A. Shaw, Christine M. Szablewski, Stephanie Kellner, Laura Kornegay, Patricia Bair, Skyler Brennan, Audrey Kunkes, Meredith Davis, Olivia L. McGovern, Jonas Winchell, Miwako Kobayashi, Nancy Burton, Marie A. de Perio, Julie Gabel, Cherie Drenzek, Julia Murphy, Caroline Holsinger, Laurie Forlano

**Affiliations:** Centers for Disease Control and Prevention, Atlanta, Georgia, USA (K.A. Shaw, C.M. Szablewski, O.L. McGovern, J. Winchell, M. Kobayashi);; Virginia Department of Health, Richmond, Virginia, USA (K.A. Shaw, S. Kellner, L. Kornegay, P. Bair, M. Davis, J. Murphy, C. Holsinger, L. Forlano);; Georgia Department of Public Health, Atlanta (C.M. Szablewski, S. Brennan, A. Kunkes, J. Gabel, C. Drenzek);; National Institute for Occupational Safety and Health, Cincinnati, Ohio, USA (N. Burton, M.A. de Perio)

**Keywords:** psittacosis, Chlamydia psittaci, bacteria, chickens, chicken slaughter plants, occupational health, disease outbreaks, respiratory infections, workers, zoonoses, Virginia, Georgia, United States

## Abstract

During August–October, 2018, an outbreak of severe respiratory illness was reported among poultry slaughter plant workers in Virginia and Georgia, USA. A multiorganizational team investigated the cause and extent of illness, determined that the illness was psittacosis, and evaluated and recommended controls for health hazards in the workplace to prevent additional cases.

Psittacosis results from inhalation of aerosolized droppings or respiratory secretions of birds infected with *Chlamydia psittaci*. During 2008–2017, a total of 60 cases of psittacosis, a nationally notifiable disease in the United States, were reported to the National Notifiable Diseases Surveillance System (https://wwwn.cdc.gov/nndss).

The most common source of psittacosis in the United States is believed to be pet psittacine birds (e.g., parrots, cockatoos). The most recent large poultry-associated outbreaks in the United States were reported 3 decades ago and were linked to turkeys (*1**,**2*). *C. psittaci* prevalence in poultry in the United States is unknown, although it has been recently identified in turkeys in the United States (*3*) and turkeys and chickens overseas (*4**,**5*). Poultry can be infected but show no overt signs of illness (*6*).

During August 31–September 4, 2018, the Virginia Department of Health (VDH) received reports of 10 persons, all workers at the same chicken slaughter plant, hospitalized with fever, headache, cough, and radiographic evidence of pneumonia. Lower respiratory tract specimens (2 bronchoalveolar lavage and 1 sputum) from 3 hospitalized workers were positive for *C. psittaci* by real-time PCR targeting the *C. psittaci* locus tag CPSIT_RS01985 (*7*), performed at the Centers for Disease Control and Prevention (CDC; Atlanta, GA, USA). The Virginia plant suspended operations on September 8.

On September 12, the Georgia Department of Public Health (GDPH) was notified that 3 employees of a Georgia chicken slaughter plant owned by the same company were hospitalized with pneumonia. *C. psittaci* was detected in sputum samples from all 3 patients. The Georgia plant suspended operations on September 15.

After plant closures, VDH and GDPH staff inspected the respective plants, which both slaughter only chickens, and collected environmental samples to test for *C. psittaci*. Staff collected samples from areas where workers were close to or directly handled live chickens or carcasses. Environmental samples were tested for chlamydial species by using real-time PCR, followed by high-resolution melt analysis (*8*), at the University of Georgia Infectious Disease Laboratory (Athens, GA, USA).

The company held employee meetings in each state and invited VDH and GDPH representatives to provide outbreak information and conduct active case finding. VDH and GDPH initiated investigations of cases and potential risk factors. A case was defined as illness in a worker employed during August 1–September 7, 2018, at the Virginia plant, or during August 13–September 28, 2018, at the Georgia plant, who had either physician-diagnosed pneumonia, or fever or chills with ≥2 symptoms of headache, cough, or muscle aches. A confirmed case required PCR detection of *C. psittaci* in a clinical specimen.

At the Virginia plant, 50 cases (including 5 confirmed) were identified; 30 cases (including 8 confirmed) were identified at the Georgia plant. PCR cycle threshold values for the 13 confirmed cases ranged from 26 to 37. Using sequencing of the outer membrane protein A (*ompA*) gene, we identified genotype D of *C. psittaci* in patient specimens; this genotype is most often found in poultry (*4**,**5*). Cases occurred during August 3–September 8 in Virginia and August 17–October 22 in Georgia.

We provide detailed characteristics for all patients (ill workers) (Table). A total of 58% of patients were men (age range 19–58 years). Bird evisceration was the most common job duty or title (reported by 53% of ill workers), consistent with previous psittacosis outbreaks (*1**,**2**,**9**,**10*). Twenty-nine workers were hospitalized (3 in intensive care) and had stays from 1 to 37 days. No deaths were reported.

**Table:** Characteristics of chicken slaughter plant workers tested during outbreak of psittacosis, Virginia and Georgia, United States, 2018

Characteristic	No. (%) workers		
	Confirmed, n = 13	Other, n = 67	All, n = 80
Sex			
M	10 (77)	36 (54)	46 (58)
F	3 (23)	31 (46)	34 (42)
Symptoms			
Fever	13 (100)	57 (85)	70 (88)
Cough	11 (85)	52 (78)	63 (79)
Muscle aches	10 (77)	47 (70)	57 (71)
Headache	9 (69)	59 (88)	68 (85)
Chills	9 (69)	51 (76)	60 (75)
Gastrointestinal*	6 (75)	13 (59)	19 (63)
Clinical			
Radiologically confirmed pneumonia	13 (100)	30 (45)	43 (54)
Hospitalized	11 (85)	18 (27)	29 (36)
Intensive care	3 (23)	0	3 (4)
Job duties†			
Evisceration	8 (62)	34 (51)	42 (53)
Live-bird handling	3 (23)	6 (9)	9 (11)
Cleaning	2 (15)	5 (7)	7 (9)
Packing or shipping	1 (8)	13 (19)	14 (18)
Inspection or quality assurance	1 (8)	6 (9)	7 (9)
Other‡	0	5 (7)	5 (6)

*Data for this symptom were not collected in Virginia; numbers reflect only Georgia numerators and denominators (confirmed, n = 8, other, n = 22; total, n = 30). †In Virginia, 1 patient did not report job duty; in Georgia, 2 patients with confirmed illness and 3 other patients reported multiple job duties. ‡Includes maintenance and human resources (plant floor–based).

*C. psittaci* was not detected in any of the environmental samples from the Virginia (n = 62) and Georgia (n = 46) plants. After extensive cleaning with sanitizers, including quaternary ammonia, chlorine solutions, and chlorine dioxide foam (all registered by the US Environmental Protection Agency as effective against *C. psittaci*), the Virginia and Georgia plants reopened on September 18 and 19, respectively. Georgia cases that occurred after the plant reopened were attributed to longer incubation periods. The incubation period for psittacosis is typically 1–4 weeks (*6*), but illness onset >30 days after exposure was reported in the 2 most recent poultry-associated outbreaks in the United States (*1**,**2*).

At the request of the US Department of Agriculture Food Safety and Inspection Service, the National Institute for Occupational Safety and Health conducted a health hazard evaluation of the Virginia plant on September 19–20. Recommendations to the plant included repositioning cooling fans, ensuring evisceration tools were working properly, and changes to other work practices to reduce bacterial contamination and aerosolization. A health hazard evaluation was not requested at the Georgia plant, but company management reported implementing or evaluating options to implement all applicable recommendations at the plants.

Clinicians evaluating poultry slaughter plant workers with febrile respiratory illness should consider psittacosis as a possible diagnosis. In the absence of a more likely diagnosis, clinicians should contact state health authorities to discuss whether *C. psittaci* testing should be requested through CDC, which has the only laboratory in the United States in which PCR testing for human specimens is currently available.
